# The Study of the Cytotoxicity, Proliferative and Microbiological Activity of the Medicated Chewing Gum with Ascorbic Acid and Lysozyme Hydrochloride Using Different Culture of Cells

**DOI:** 10.3390/pharmaceutics15071894

**Published:** 2023-07-05

**Authors:** Yuliia Maslii, Liudmyla Garmanchuk, Olena Ruban, Taisa Dovbynchuk, Nataliia Herbina, Giedre Kasparaviciene, Jurga Bernatoniene

**Affiliations:** 1 Department of Industrial Technology of Drugs, National University of Pharmacy, 61002 Kharkiv, Ukraine; yuliia.maslii@lsmu.lt (Y.M.); ruban_elen@ukr.net (O.R.); nataliia.herbina@lsmu.lt (N.H.); 2 Department of Drug Technology and Social Pharmacy, Faculty of Pharmacy, Medical Academy, Lithuanian University of Health Sciences, LT-50161 Kaunas, Lithuania; giedre.kasparaviciene@lsmu.lt; 3 Institute of Biology and Medicine, Taras Shevchenko National University of Kyiv, 01601 Kyiv, Ukraine; liudmyla_garmanchuk@ukr.net (L.G.); mtaisa80@gmail.com (T.D.); 4 Institute of Pharmaceutical Technologies, Faculty of Pharmacy, Medical Academy, Lithuanian University of Health Sciences, LT-50161 Kaunas, Lithuania

**Keywords:** medicated chewing gum, ascorbic acid, lysozyme hydrochloride, preclinical study, microbiological research, cultures of cell

## Abstract

Medicated chewing gum with lysozyme hydrochloride and ascorbic acid as active pharmaceutical ingredients was developed for application in dentistry. The aim of this research was to study the cytotoxicity, proliferative, and microbiological activities of the active ingredients in different types of cell cultures. The preclinical study of active pharmaceutical ingredients and their combinations was carried out using culture lines such as HepG2 (human hepatocarcinoma cells), Hek293 (human embryonic kidney cells), and MAEC (mouse aortic endothelial cells). MTT assays were used to analyse cytotoxicity and proliferative activity, while the state of antioxidant protection was assessed by the content of sulfhydryl groups and catalase activity. The determination of lipid peroxidation products was based on the level of TBA-active products. As a microbiological model for studying the effect of the developed dental medicine on the ability of the oral cavity microorganisms to form biofilms, the following strains were used: *Streptococcus mutans*, *Staphylococcus aureus*, *Staphylococcus epidermidis*, *Lactobacillus plantarum*, and *Candida albicans*. The optical density of the formed biofilm was evaluated by the intensity of the experimental sample’s colour on a StatFax 303 Plus photometer at a wavelength of 630 nm. The combination of ascorbic acid and lysozyme hydrochloride in the established concentrations (20 mg and 10 mg per 1 gum, respectively) resulted in a slight stimulation of cell proliferation without any toxic effects and increased antioxidant protection, preventing the development of oxidative stress. It was found that, in contrast to the separately used active substances, the combination of lysozyme hydrochloride and ascorbic acid inhibits the biofilm formation of all studied microorganisms and shows the ability to destroy diurnal biofilms of *L. plantarum* and fungi of the genus *Candida*, indicating potentiation and summation of the active pharmaceutical ingredients’ composition effects in the developed dental medicine. Due to the observed positive pharmacological and microbiological action, the combination of lysozyme hydrochloride and ascorbic acid in the medicated chewing gum serves as a promising tool for the prevention and treatment of infectious and inflammatory diseases of the periodontium and mucous membranes and the prevention of caries.

## 1. Introduction

**Figure 1:**
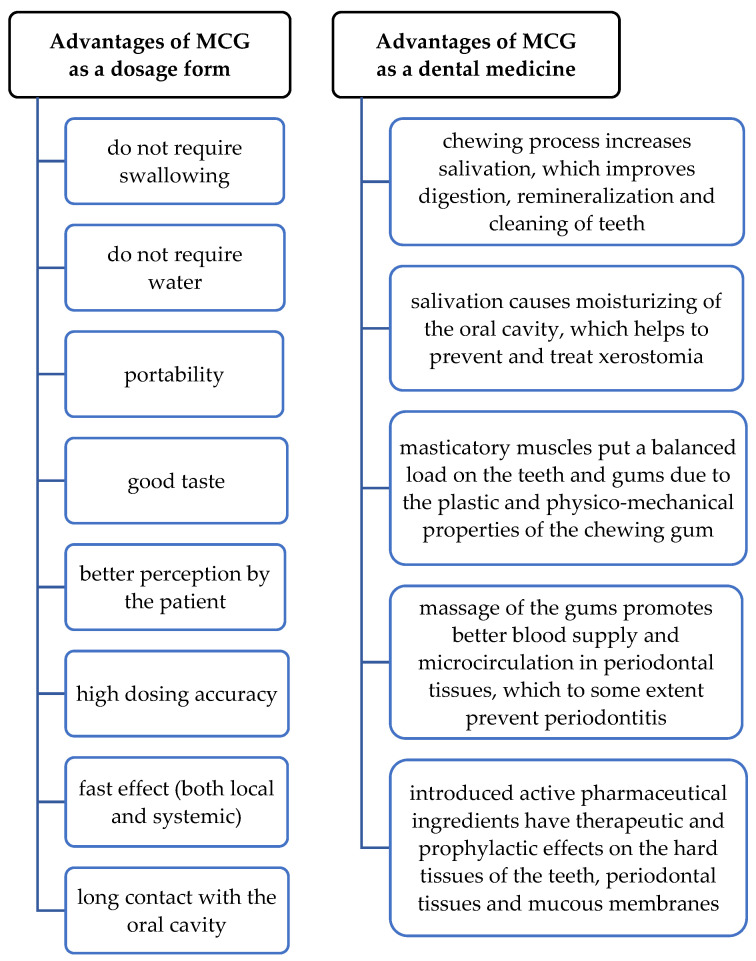
Advantages of medicated chewing gum as a dosage form and as a dental medicine.

Compressed medicated chewing gum (MCG) is a promising and convenient dosage form of delivering drugs for the prevention and treatment of dental diseases [1,2,3,4]. When chewed, it releases active substances directly in the place of action and has a positive effect on the hard and soft tissues of the oral cavity (Figure 1).

In our previous investigations, we have substantiated the composition and the method of preparation of compressed medicated chewing gums, which were intended for the prevention and treatment of dental diseases, inflammatory diseases of the periodontium (gingivitis, periodontitis), mucous membranes (stomatitis), caries, and manifestations of xerostomia in particular [5]. Lysozyme hydrochloride and ascorbic acid were chosen as active pharmaceutical ingredients.

Lysozyme is a glycoside hydrolase that catalyses hydrolysis of β1→4 bonds between N-acetylmuramic acid residues and N-acetyl-D-glucosamine in peptidoglycan, which is a major component of the Gram-positive bacterial cell wall. The hydrolysis disrupts the integrity of bacterial cell walls, causing bacterial lysis. Moreover, lysozyme can inactivate a layer of non-antigenic glycosaminoglycans in tumour cells and thus inhibit their growth [6,7,8,9,10]. In addition to antimicrobial action, lysozyme hydrochloride serves as a protective agent against caries by violating the ability of microorganisms to be attached to a tooth surface [11] and has anti-inflammatory and immunomodulating effects [12,13].

The important role of vitamin preparations in the complex therapy of dental diseases is well known. Ascorbic acid deficiency has been proven to cause dryness of the oral mucosa, gingival bleeding, and petechial haemorrhages in various parts of the mouth, resulting in xerostomia, ulcerative gingivitis, and stomatitis development [14,15]. In addition, it was established that vitamin C at low concentrations can be used to effectively disrupt bacterial biofilm formation, which is the cause of the emergence and development of many dental pathologies [16]. Ascorbic acid is a naturally occurring organic compound and a potent antioxidant, preventing oxidative damage to lipids and other macromolecules. It neutralizes superoxide radicals into hydrogen peroxide and stimulates interferon synthesis [17]. Ascorbic acid can also exhibit bimodal activity, switching from an antioxidant in low-concentration physiologic conditions to a pro-oxidant under pathologic conditions [18,19]. Ascorbic acid is involved in the synthesis of collagen, serotonin from tryptophan, the synthesis of catecholamines and the formation of corticosteroids [20,21]. It is involved in the conversion of cholesterol into bile acids [22]. Detoxification in hepatocytes also occurs with the participation of cytochrome P450 and ascorbic acid [23,24].

Therefore, the combination of lysozyme hydrochloride and ascorbic acid in MCG composition will provide a complex effect on the tissues of the oral cavity: antimicrobial, anti-inflammatory, local immunomodulatory, antioxidant, and antiviral; it will enhance tissue regeneration and epithelialization; regulate blood clotting and normalize capillary permeability; stimulate salivation; and prevent plaque. In addition to this wide range of effects on the tissues of the masticatory and speech apparatus, ascorbic acid gives a pleasant taste to the developed gum.

According to the literature, the amount of lysozyme hydrochloride in one dose of various oral preparations (Lysobact^®^, Bosnalijek, Sarajevo, Bosnia and Herzegovina; Lysopain^®^ N, Sanofi-Aventis, Visp, Switzerland; Glossithiase^®^, Jolly-Jatel, Saint-Germain-en-Laye, France; etc.) varies from 5 to 20 mg, while the dose of ascorbic acid, according to the recommendations of the European Commission’s Council on nutrition labelling, is 80 mg/day [25]. It is known that dentists recommend using chewing gums no more than two to three times a day after meals; therefore, the suggested concentrations of 10 mg of lysozyme hydrochloride and 20 mg of ascorbic acid per gum do not exceed these doses.

Previous studies focused on the uniformity evaluation of the obtained MCGs [5] (according to the European Pharmacopoeia (Ph. Eur.) 9.0 Chapters 2.9.5, 2.9.6, and 2.9.40 [26]) and their mechanical, textural, release (Ph. Eur. 9.0 Chapter 2.9.25 [26]) and chewing perception characteristics [27]. The propriety of the MCG preparation method involving the wet granulation step has been confirmed by mass and drug content uniformity tests [5]. The optimal compressing force was selected to be 7 kN, which allowed to obtain MCGs with good organoleptic, mechanical, textural, and release properties. Good release profiles of active pharmaceutical ingredients from the developed chewing gums were determined—“chewing” for 30 min led to almost complete their dissolution: 99.52 ± 0.33% of lysozyme hydrochloride and 98.31 ± 0.52% of ascorbic acid, which indicates their high bioavailability [27]. A somewhat lower result of ascorbic acid release can be explained by its tendency to get oxidized and, accordingly, its possible minor metabolism in the oral cavity.

The biotope of the oral cavity Is characterized by a large number (more than 700 species) of microorganisms that create dental plaque, which, according to recent studies, should be considered as a biofilm [28]. The emergence and spread of infectious and inflammatory diseases of the oral cavity and pathologies of the teeth’s hard tissues are closely related to the ability of pathogenic microorganisms to adhere and form biofilms [29]. Therefore, the problem of overcoming the ability of bacteria to form biofilms is one of the urgent tasks of modern antimicrobial therapy. Among the promising ways to solve this problem is the search for antimicrobial agents capable of both preventing the formation of biofilms and ensuring their destruction.

In addition, the application of alternative methods of pharmacological screening of potential medicines for medicinal suitability is a key stage of drug development. Numerous achievements of fundamental and applied research in biology and medicine in recent decades are associated with the use of cultured cells of various genesis as sensitivity models.

Therefore, the aim of the present study was to determine the action effectiveness of the active pharmaceutical ingredients of the dental medicine in the form of compressed MCG, namely ascorbic acid and lysozyme hydrochloride, by microbiological and pharmacological evaluation using different culture of cells. For the study of proliferative, cytotoxic or metabolic effects, the embryonic cells, endothelial cells, and cell lines of hepatocytic origin were used. Since oral streptococci, opportunistic staphylococci, Candida fungi, and lactobacilli play a special role in the formation of dental plaque [30], strains of the following microorganisms were used as microbiological models: *Streptococcus mutans (S. mutans)*, *Staphylococcus aureus (S. aureus)*, *Staphylococcus epidermidis (S. epidermidis)*, *Lactobacillus plantarum (L. plantarum)*, and *Candida albicans (C. albicans).*

## 2. Materials and Methods

### 2.1. Materials

The list of active pharmaceutical ingredients and excipients and their functions in the MCG formulation is presented in Table 1.

### 2.2. Medicated Chewing Gums Preparation

MCG’s preparation technology was as follows: powders of lysozyme hydrochloride (Bouwhuis Enthoven B.V., Raalte, The Netherlands), apple taste additive (Kerry Inc., Kuala Lumpur, Malaysia), and sucralose (V.B. Medicare Pvt. Ltd., Karnataka, India) were dry-blended, wetted with ethyl alcohol 96%, then granulated through a sieve and dried at room temperature. The obtained granules were thoroughly mixed with ascorbic acid (Foodchem International Corporation, Shangai, China), Health in Gum^®^ PWD 01 (Cafosa Gum SA, Barcelona, Spain), and Syloid^®^ FP244 (Grace GmbH & Co. KG, Worms, Germany), on which the Apple liquid flavour (Kerry Inc., Kuala Lumpur, Malaysia) was previously sprayed. At the last stage, the homogeneous mass of magnesium stearate (S.D. Fine Chemicals Ltd., Mumbai, India) was added to this mixture. Round flat-faced chewing gums with a mass of 1000 mg and a diameter of 13 mm were compressed at a compression force of 7 kN on a laboratory single-punch tablet machine (model HTM-01E, Mariupol Plant of Technological Equipment, Mariupol, Ukraine) [27].

### 2.3. Dissolution Test for Medicated Chewing Gum

The research was conducted in vitro according to the Ph. Eur. “Dissolution test for medicated chewing gums” (2.9.25) using a special apparatus that simulates the chewing process (type B) [26]. Dissolution parameters: chewing frequency—60 cycles/min, distance between surfaces—1.4 mm, the number of test gum samples—6. One gum was placed in the device, and 20.0 mL of a phosphate buffer solution with pH 6.0 R2 was used as a chewing medium. After 30 min of “chewing”, 2.0 mL aliquots were taken for testing. The selected samples were studied using the developed physicochemical methods of active pharmaceutical ingredients analysis: titrimetric determination of ascorbic acid and spectrophotometric determination of the lysozyme hydrochloride content [27].

Before adding to the cells, aliquots containing the components under study were sterilized by filtering through 0.22-micron nitrocellulose filters.

### 2.4. Cell Culture and Cultivation Conditions

The following cell lines were used in the experiment: HepG2—human hepatocarcinoma cells (85011430 Sigma ECACC Cell Line; Sigma, St. Louis, MO, USA), Hek293—human embryonic kidney cells (85120602 Sigma ECACC Cell Line; Sigma, St. Louis, MO, USA), and MAEC (mouse aortic endothelial cells). Cultivation was performed in plastic Petri dishes and 96-well plate (Orange Scientific, Braine-l’Alleud, Belgium). DMEM medium (Sigma, St. Louis, MO, USA) with 10% foetal calf serum (FCS) (Sigma, St. Louis, MO, USA) or RPMI-1640 with HEPES, and 80 μg/mL gentamicin was used for cultivation. Cultivation was performed in a CO_2_ incubator at 95% humidity, 5% CO_2_ content, and a temperature of (37 ± 1) °C.

The action of active pharmaceutical ingredients was determined in comparison with the control (cells without tested compounds).

### 2.5. Determination of Cytotoxicity/Proliferative Activity

The determination of cytotoxicity/proliferative activity under the influence of test compounds was performed on the cells Hek293 using the MTT assay [31,32,33]. As studied samples containing the tested active pharmaceutical ingredients in the required concentrations, prepared aliquots obtained after MCGs dissolution were used. Under the above conditions, plates with cells with the addition of different concentrations of tested substances were incubated for 24 h, and in control wells was medium without tested substances (negative control). MTT-reagent (a colourless salt of tetrazolium (Sigma Chemical Co., St. Louis, MO, USA)) was introduced into the wells to the cells at 20 μL up to a final concentration of 0.6 mM and incubated for 4 h in a CO_2_ incubator under conditions favourable for the reduction by cells of soluble MTT-reagent to insoluble formazan. After this time, the medium was removed from the wells, and 100 μL of dimethyl sulfoxide was added to solubilize formazan. The plate was gently shaken for 5–10 min until the formazan crystals dissolved [34,35,36].

Using a plate reader (BioTek, Shoreline, WA, USA), the optical density of each well at 540 nm was determined, subtracting the measured background absorption at 620 nm. The results were calculated by the Formula (1) and given as a percentage of the values obtained for the control sample [37,38]:

RAU = optical density of the sample with cells − optical density of the sample without cells (1)

The calculation of the live and dead cell concentration ratio was carried out by routine counting in the Goryaev chamber after staining the dead cells with trypan blue.

### 2.6. Determination of TBA-Active Products Content and Accumulation in Cells

The ascorbic acid and lysozyme hydrochloride effects on the performance of the antioxidant system have been determined in cells HepG2. To induce oxidative stress in cells, a 1 mM H_2_O_2_ solution was used. To do this, part of the cells that reached 80% confluent was incubated for 4 h with hydrogen peroxide, after which ascorbic acid, lysozyme hydrochloride, and their combination, contained in aliquots obtained after MCGs dissolution, were added. The other half of the cells were incubated without the addition of hydrogen peroxide.

The concentration of TBA-active products was determined by the method of I. D. Stalna and T. G. Garishvili (1977) by reaction with 2-thiobarbituric acid (TBA) [39]. The samples were homogenized in saline. Subsequently, 400 μL of 20% trichloroacetic acid was added to all tubes and centrifuged for 15 min at 1000 rpm. 0.5 mL of 0.8% TBA was added to the supernatant and incubated for 15 min at 100 °C in a water bath. The determination of the optical density of the samples at λ = 532 nm was performed on a spectrophotometer (BioTek, Shoreline, WA, USA).

The content of TBA-active products per 1 mg of protein was calculated based on the value of the molar extinction coefficient of the complex of malonic dialdehyde with 2-thiobarbituric acid.

### 2.7. Determination of Catalase Activity

Catalase activity in cells was calculated by comparing the hydrogen peroxide content in the sample at the beginning of the reaction and after it [40,41]. For this purpose, two samples were prepared: zero and experimental (aliquots obtained after MCGs dissolution). The cell lysate was prepared by homogenization in saline (Hanks’ buffer/0.05 M Tris-HCl (pH = 7.8)) in a ratio of 1:3. 0.5 mL of reaction buffer containing 0.03% H_2_O_2_ was added to 25 μL of protein extract (0.025 mg of protein) and incubated for 10 min at room temperature. The reaction was stopped by adding 0.5 mL of a 4% ammonium molybdate ((NH_4_)_2_Mo_7_O_24_) solution. The mixture with 0.025 mL of distilled water instead of protein was incubated with the reaction mixture for 10 min at room temperature, and the reaction was stopped by adding 0.5 mL of a 4% (NH_4_)_2_Mo_7_O_24_ solution (zero point). The colour intensity was measured on a reader (BioTek, Shoreline, WA, USA) at λ = 410 nm against the control sample (zero point).

### 2.8. Determination of the Level of Total, Protein-Bound and Non-Protein Sulfhydryl (SH) Groups

The level of total, protein-bound, and non-protein SH-groups was measured by the Elman method [42]. The determination of the total content of SH-groups was performed by adding 0.5 mL of 30 mM Tris-hydrochloride (Tris-HCl) with 1 mM ethylenediaminetetraacetic acid (EDTA) (pH = 8) to 0.075 mL of cell homogenate, obtained after incubation of cells with an aliquot after MCGs dissolution, and adding 0.05 mL of 1.25% sodium dodecyl sulphate (SDS). The mixture was incubated for 15 min at room temperature. Next, 0.025 mL of Elman’s reagent was added for detection and incubated for 30 min in the dark. Extinction was measured at λ = 412 nm against a control sample (0.575 mL of 30 mM Tris-HCl with 1 mM EDTA with the addition of 0.05 mL of 1.25% SDS and 0.025 mL of Elman’s reagent).

The determination of the non-protein SH-groups content was performed by adding 0.025 mL of 10.5% trichloroacetic acid to 0.075 mL of homogenate. The samples were incubated for 10 min at room temperature, followed by centrifugation for 15 min at 1500 rpm. The supernatant was neutralized by adding 1 M NaOH to pH = 7.0. For detection, 0.525 mL of 30 mM Tris-HCl with 1 mM EDTA (pH = 8) and 0.025 mL of Elman’s reagent were added and incubated for 30 min in the dark. Extinction was measured at λ = 412 nm against the control sample (0.625 mL of 30 mm Tris-HCl with 1 mM EDTA and the addition of 0.025 mL of Elman’s reagent).

The determination of the protein SH-groups content was performed by subtracting the non-protein SH-groups content from the total content of SH-groups Formula (2):

C (protein) = C (total) − C (non-protein)(2)

### 2.9. Study of Morphological Features of Cells

To determine the morphological characteristics of cells under the influence of active substances, mouse aortic endothelial cells of the MAEC line obtained from the collection of the company “Sigma” (Sigma-Aldrich, St. Louis, MO, USA) were used as described by us earlier [43].

To cells that were in 50–60% of the monolayer, 20 mg/mL of each of the studied compounds, contained in aliquots after MCGs dissolution, was added separately and in combination. After 24 h of cultivation, the culture medium was removed, the cells were washed twice with phosphate-buffered saline, and they were fixed with 95% ethanol for 30 min. Then ethanol was removed, and the cells were dried and stained with crystal violet dye for 20 min at a temperature of 37 °C. Assessment of morphological parameters and visualization of cell populations were performed using an inverted microscope AxioVert (Carl Zeiss, Oberkochen, Germany) equipped with AxioVision Rel. 4.8.2 software (Carl Zeiss AG, Jena, Germany). Cellular preparations were photographed using a Digital Still Camera with a Carl Zeiss Vario-Sonar lens.

### 2.10. Study of the APIs Effect on Biofilm Formation

To study the ability of strains to form biofilms, their pure cultures were sown on nutrient agar and incubated in a thermostat for 24 h at 37 °C (*S. mutans*, *S. aureus*, *S. epidermidis*, and *L. plantarum*) and 25 °C (*C. albicans*). Washout from the agar culture was carried out by adding 1 mL of isotonic sodium chloride solution and bringing it to the turbidity standard, considering the amount of 10^9^ cells/cm^3^.

Sterile flat-bottomed 96-well plates were used to detect biofilm formation. Each well was filled with 0.2 mL (200 μL) of the prepared suspension with the aliquots after MCGs dissolution that contained the studied substances individually and in combination (3 wells for each strain). As a control, only the appropriate nutrient medium is used to check for sterility and non-specific binding of the nutrient medium components to the tablet.

Tablets were incubated for 4, 24, 48, and 72 h at 35 °C. After incubation, the contents of the wells were removed, and the wells were washed 4 times with 0.2 mL of phosphate-salt buffer (pH 7.2) to eliminate free-living “plankton” bacteria. The formed biofilms were fixed with a 2% solution (volume fraction) of sodium acetate and stained with a 0.1% solution of crystal violet for 30 min at room temperature. The excess dye was removed, the wells were washed three times with distilled water, and then the tablets were air-dried (30 min). To extract the dye, 0.2 mL of 96% ethanol was added to the wells and left for 60 min at room temperature [44,45]. The optical density (O.D.) of the formed biofilm was estimated by the intensity of alcohol staining on a photometer StatFax 303 Plus (Awareness Technology, Palm City, FL, USA). Since clear wavelength parameters for evaluating the formed biofilm have not been established to date, in our comparative studies we used a wavelength of 630 nm (maximum), which, according to the literature, is optimal for measurements when using gentian violet dye [46].

The obtained optical density values were taken as an index of bacterial adhesion to the surface and the ability to biofilms formation. The parameters given in the Table 2 were used to evaluate the obtained results [47].

### 2.11. Statistical Analysis

Statistical processing of the obtained results was performed using the program “STATISTICA 8.0”. The probability of differences between the control and experimental groups was determined by the criteria of Student and Fisher. The level of reliability was taken at *p* < 0.05.

## 3. Results and Discussion

Alternative methods of determining the pharmacological action of potential medicines involve their cytotoxic/cytostatic screening on a panel of cultured cells with various sensitive markers for target exposure. In our experimental studies of the constituent components that saturate the MCG, the following cell lines were selected. Hek293 is a cell line of embryonic human kidney origin that mainly consists of endothelial, epithelial, and fibroblast cells. These cells are sensitive to cytotoxic effects and are therefore widely used in test systems in original studies of promising pharmacological drugs [48]. When studying the systemic metabolism of drugs, cell lines of hepatocyte origin are used, such as the HepG2 cell line [49,50,51], which we used to determine the possible risks of the applied combination of ascorbic acid and lysozyme hydrochloride on SH-groups, TBA-active components, and catalase activity as important indicators of antioxidant protection. MAEC line was used as a model system that plays an important role in the endothelial barrier of drug permeability [52,53] to determine possible changes in the morphofunctional features of these cells under the action of ascorbic acid and lysozyme hydrochloride.

The aliquot samples obtained after the dissolution test with appropriate concentrations of active pharmaceutical ingredients were used in further studies of anti/proliferative effects.

### 3.1. Study of the Cytotoxic Effect of Active Pharmaceutical Ingredients on Human Embryonic Kidney Cells of the Hek293 Line

Determination of cytotoxicity/proliferative activity relative to cells of embryonic origin Hek293 under the influence of lysozyme hydrochloride and ascorbic acid was performed using an MTT assay. The MTT colorimetric assay is based on the ability of mitochondrial enzymes to convert the salt of 3-(4,5-dimethylthiazol-2-yl)-2,5-diphenyltetrazolium bromide (monotherazolium salt) yellow colour into crystals of formazan purple colour, the intensity of which allows to determine the cytotoxicity/proliferation of cultured cells. Mitochondrial succinate dehydrogenase and cytochrome C are mainly involved in MTT reduction. Thus, compounds that affect the intensity of MTT reduction can act as mitochondrial respiration modifiers and, accordingly, can be used to determine viable cells.

In order to study the effect of the studied active pharmaceutical ingredients on cells and to confirm the proposed concentrations, in this experiment, lysozyme hydrochloride and ascorbic acid were added to the composition of MCG in a wider range of concentrations—1–40 mg/per gum.

**Figure 2:**
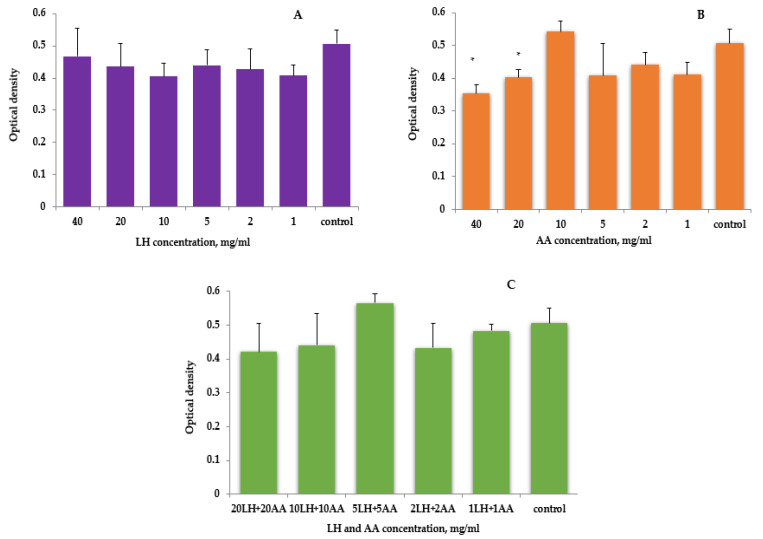
The effect of active pharmaceutical ingredients in the concentration range of 1–40 mg/mL on Hek293 cells (RPMI-1640 environment with HEPES): (**A**)—lysozyme hydrochloride (LH); (**B**)—ascorbic acid (AA); (**C**)—lysozyme hydrochloride and ascorbic acid combination (LH + AA), *—*p* < 0.05 vs. control, *n* = 3.

As presented by the results of active pharmaceutical ingredients’ effects on human embryonic cells of the Hek293 line, lysozyme hydrochloride has not shown toxicity in the entire studied range of concentrations—the relative values of cell viability in comparison with the control were in the range of 79.87–92.43%; however, these data were not statistically significant (Figure 2A).

When ascorbic acid concentrations of 1 to 20 mg/mL were used, no toxic effects were detected, but inhibition of proliferation was—the relative values of cell viability from the control were in the range of 79.3–106.8%. When ascorbic acid was added to the cells (Figure 2B), the optical absorbance determined in the MTT test decreased 1.3 times compared to the control, but this effect was cytostatic. Thus, when counting cells with trypan blue, the concentration of cells was (12.7 ± 1.4) thousand against (16.4 ± 2.1) thousand in the control, but the percentage of dead cells did not differ from the control and was 5.7 ± 2.4%. Therefore, the maximum amount of active pharmaceutical ingredient needed to study their combined effect on the Hek293 cell line was 20 mg/mL.

The combined use of lysozyme hydrochloride and ascorbic acid in the range of studied concentrations also did not lead to a decrease in the number of viable cells (Figure 2C).

However, this could not be said about the direct toxic effect of ascorbic acid on the studied cells—the inhibition of proliferation by higher concentrations can be explained by a shift of pH to the acidic side (≈4), because acidification of the medium did not allow normal cell regeneration (the optimal pH value of the culture medium should be in the range of 6.8–7.2 [54]). Thus, the determination of cell viability by counting living and dead cells after staining with trypan blue was about 15 ± 4%, while in the control this figure was 11 ± 2%, which is statistically insignificant.

The results correlate with the experimental data of other scientists, who determined that the concentrations of ascorbic acid at 30 mg and 60 mg were too low to adversely affect the hard tissues of the teeth, with a positive effect on bleeding gums and a significant reduction in tartar and plaque after the use of chewing gum with ascorbic acid at these concentrations [55]. In addition, our biopharmaceutical studies confirmed a slight shift in pH (from 6.07 to 5.70) after “chewing” gums for 10 min [27].

Therefore, testing the combination of lysozyme hydrochloride and ascorbic acid as components of MCG on cells of embryonic origin of the Hek293 line proved the effectiveness of these compounds: they did not show toxic antiproliferative effects but slightly stimulated cell proliferation, which would have a positive effect on the cells of the oral cavity in destructive-inflammatory diseases of the periodontium and mucous membranes.

### 3.2. Study of the Enzyme Activity of the Antioxidant System in the Culture Medium of Cells under the Action of Ascorbic Acid and Lysozyme Hydrochloride

In the pathogenesis of periodontal tissues inflammatory diseases, a significant role is played by prooxidant-antioxidant homeostasis disorder, which is manifested by activation of lipid peroxidation and reduced enzymes activity of antioxidant protection [56,57,58].

Among the main representatives of reactive oxygen species in a live cell, superoxide anion radical ($O_{2}^{•-}$), hydroxyl radical (OH•), and hydrogen peroxide (H_2_O_2_) are distinguished. Hydrogen peroxide is the least toxic and longest-lived (10^−2^ s) active oxygen metabolite and belongs to medium-strength oxidants. The generation of H_2_O_2_ occurs mainly on the nuclear, plasma, and mitochondrial membranes. Hydrogen peroxide easily penetrates cell membranes and is able to diffuse in the cell over considerable distances. There is an antioxidant defence system in the body to prevent the excess formation of reactive oxygen species, neutralize their damaging effects, and maintain cellular homeostasis [59].

The components of this system are low- and high-molecular compounds that are capable of intercepting oxygen free radicals, turning them into less harmful compounds, or neutralizing the source of their occurrence. Enzymatic antioxidants contain metals with variable valency in the active centre and are characterized by high specificity for certain reactive oxygen species. One of the antioxidant system’s representatives is catalase [60].

HepG2 cells of hepatocyte origin were used to study the enzyme activity of the antioxidant system. The main indicators that were studied as the most important are the level of TBA-active products, SH-groups, and catalase activity. In this analysis, fixed concentrations of active pharmaceutical ingredients were used: 10 mg of lysozyme hydrochloride and 20 mg of ascorbic acid, which correspond to their amounts in the developed MCG.

In the case of increased intake of xenobiotics, depletion of antioxidant depots, malnutrition, and other negative effects, oxidative stress occurs, which is characterized by prooxidant and antioxidant balance disorders with the predominance of the first and the development of oxidative damage. The state of antioxidant protection was assessed by the content of SH-groups and the activity of one of the most important enzymes of the antioxidant system—catalase, whose physiological role is to protect organism against exogenous and endogenous oxidative stresses by neutralizing free oxygen radicals [61].

**Figure 3:**
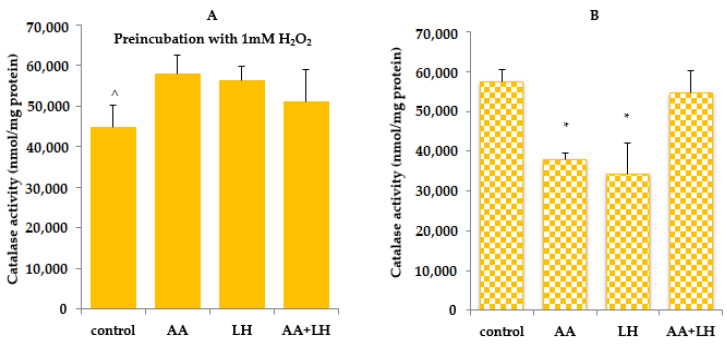
The effect of ascorbic acid (AA), lysozyme hydrochloride (LH), and their combination (AA + LH) on catalase activity with (**A**) and without (**B**) the action of hydrogen peroxide, *—*p* < 0.05 vs control, ^—*p* < 0.05 vs combination of both active substances (LH + AA), *n* = 3.

**Figure 4:**
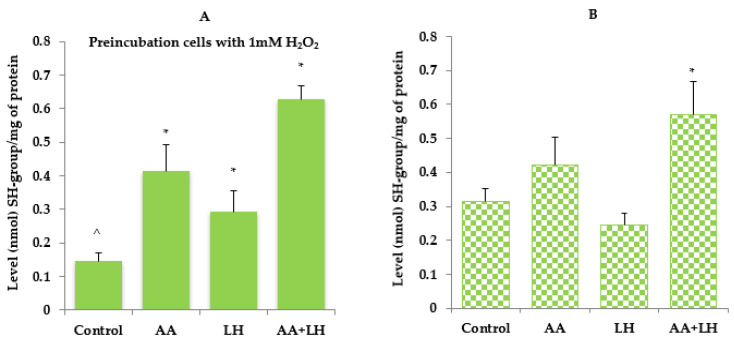
The effect of ascorbic acid (AA), lysozyme hydrochloride (LH), and their combination (AA + LH) on the level of SH-groups in the cell culture medium with (**A**) and without (**B**) the action of hydrogen peroxide, *—*p* < 0.05 vs. control, ^—*p* < 0.05 vs. combination of both active substances (LH + AA), *n* = 3.

**Figure 5:**
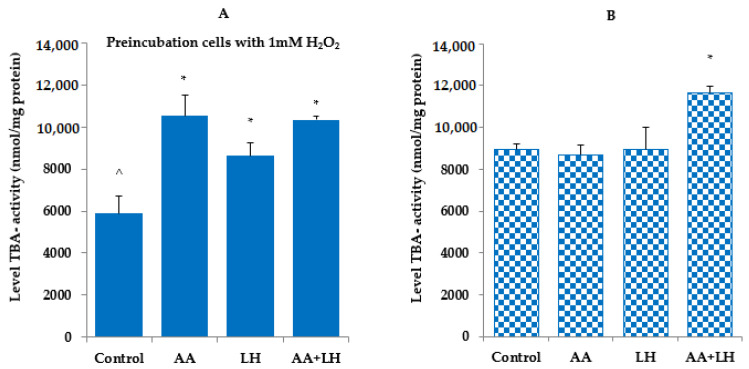
The effect of ascorbic acid (AA), lysozyme hydrochloride (LH) and their combination (AA + LH) on the level of TBA-active products in the cell culture medium with (**A**) and without (**B**) the action of hydrogen peroxide, *—*p* < 0.05 vs. control, ^—*p* < 0.05 vs. combination of both active substances (LH + AA), *n* = 3.

The obtained data indicate that in cells exposed to hydrogen peroxide, the level of TBA-active products, SH-groups, and catalase activity were lower compared to controls without exposure to hydrogen peroxide (Figure 3, Figure 4 and Figure 5). The addition of ascorbic acid, lysozyme hydrochloride, and their combination to hydrogen peroxide-induced cells increased the level of all the investigated indicators, which was commensurate with the control without preincubation with hydrogen peroxide. The most normalizing effect was recorded when using a combination of ascorbic acid and lysozyme hydroxide in concentrations that were equal to their content in chewing gum.

As shown by the results obtained without the action of hydrogen peroxide (Figure 3B), catalase activity under the influence of individual substances in comparison with the control decreased by 30% in lysozyme hydrochloride and by 18% in ascorbic acid. The enzyme activity under the action of the combination of both active substances was almost unchanged compared to the control. Therefore, the combination of lysozyme hydrochloride and ascorbic acid completely inhibited the outbreak of lipid peroxidation and restored antioxidant protection, in contrast to the use of these substances separately. Upon induction by hydrogen peroxide, the catalase activity increased under the action of lysozyme hydrochloride, ascorbic acid, and their combination and reached the activity values of this enzyme in the control with the induction of oxidative stress by hydrogen peroxide (Figure 3A).

In cells, without the action of hydrogen peroxide, the level of SH-groups, which is a quantitative characteristic of one of the main water-soluble antioxidants, reduced glutathione, slightly decreases under the action of lysozyme hydrochloride compared to the control, while ascorbic acid in mono-effect and in combination with lysozyme hydrochloride increases the level of SH-groups by 35% and 83%, respectively (Figure 4B). The increase in SH-groups activity in the culture medium of the control sample indicated the probable depletion of this link of antioxidant protection. The use of the lysozyme hydrochloride and ascorbic acid combination has been shown to improve protection against lipid peroxidation and to prevent the development of oxidative stress, which has pathogenetically substantiated the active pharmaceutical ingredients used for the prevention and treatment of inflammatory periodontal diseases. Under conditions of induction by hydrogen peroxide of oxidative stress in cells, the level of SH-groups is twice as low as in the corresponding control (Figure 4A). However, under the action of the studied components in monoapplication and in combination, it increased compared to the control induced by hydrogen peroxide. Thus, lysozyme hydrochloride increased the level of SH-groups almost twice, ascorbic acid almost three times, and the combination of these active ingredients led to a four-fold increase in SH-groups (Figure 4A).

As hydroperoxides are not stable, their decomposition leads to the appearance of various secondary and final products of lipid peroxidation, which are highly toxic compounds (diene conjugates, malonic dialdehyde, and Schiff bases), causing damaging effects on membranes and cell structures. A simple and available method for determining lipid peroxidation products is the determination of TBA-active products [62].

TBA-active products always have a constant content in tissues and cells, but with pathologies, their level can either increase or decrease. In the study of TBA-active products, it was found that their level under the action of lysozyme hydrochloride and ascorbic acid (both individually) did not differ from the control (Figure 5B), while under the combined effect of lysozyme hydrochloride and ascorbic acid, an increase in TBA-active products of 30% was revealed compared to the control. Induction of oxidative stress by hydrogen peroxide caused a 52% decrease in TBA-active products compared to the corresponding control without hydrogen peroxide (Figure 5A), and the addition of ascorbic acid, lysozyme hydrochloride, and their combination enhanced the level of TBA-active products by 78%, 46%, and 75%, respectively, compared to the corresponding control (under the action of hydrogen peroxide).

So, it can be argued that ascorbic acid and lysozyme hydrochloride balance the antioxidant defence and inhibit lipid peroxidation.

### 3.3. Study of the Active Pharmaceutical Ingredients Effect on the Morphological Characteristics of Mouse Aortic Endothelial Cells

**Figure 6:**
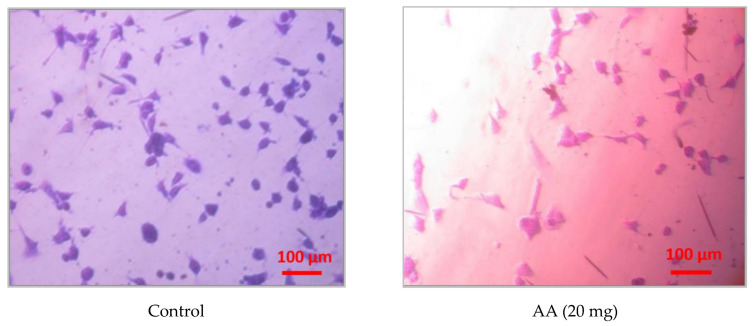

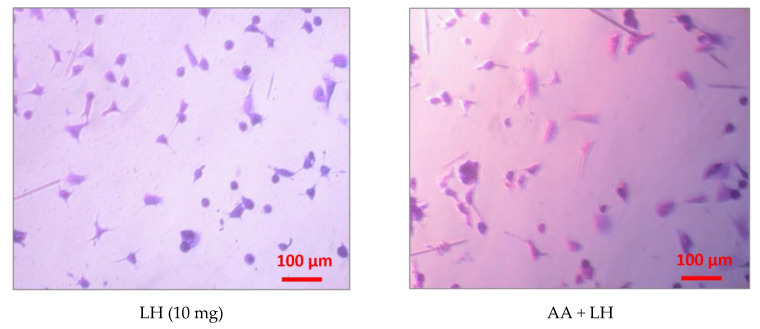
Microphotographs of endothelial cells (MAEC lines), stained with crystal violet, under the action of lysozyme hydrochloride (LH) and ascorbic acid (AA), separately and in combination.

Figure 6 shows microphotographs of endothelial cells (MAEC lines), stained with crystal violet, under the action of lysozyme hydrochloride and ascorbic acid in the concentrations, selected in the MCG composition: 10 mg and 20 mg, respectively.

As the results showed, the degree of MAEC cells spreading on the surface of the Petri dish under the influence of lysozyme hydrochloride and ascorbic acid did not differ from the corresponding control. However, the addition of ascorbic acid separately and in combination with lysozyme hydrochloride changed the colour of the endothelial cells to bright pink, in contrast to the control, where the colour of the cells was purple, which is characteristic of this dye. Violet staining of MAEC cells was also observed when only lysozyme hydrochloride was administered. At the same time, no morphological changes were observed, and the number of dead cells did not increase. The change in the colour of the dye can be explained by a shift in the pH of the environment to the acidic side due to the penetration of ascorbic acid into the cells.

Therefore, the conducted studies proved the absence of cytotoxic effects of active pharmaceutical ingredients on MAEC cells, which confirms the data obtained regarding the effect of the lysozyme hydrochloride and ascorbic acid combination on cells of other origin.

A potential limitation of this manuscript is that it was only an in vitro study, as an in vivo test could provide a clearer understanding of the effects of the API in the medicated chewing gum composition on periodontal and mucosal tissues. In addition, specific types of cell lines were used in the study, such as HepG2, Hek293, and MAEC. In further studies, it is planned to conduct additional experiments using periodontium and mucous membrane-derived cells to conclude about the positive role of the combination of lysozyme hydrochloride and ascorbic acid in the prevention and treatment of destructive and inflammatory periodontal diseases and mucous membranes. In this pre-screening study on the combined effect of the medicated chewing gum components in the therapeutic range on cell cultures, no cytotoxic/cytostatic effect of these components was recorded. It is assumed that in the prevention and treatment of destructive and inflammatory diseases of the periodontium and mucous membranes, these components will not cause side effects. However, to confirm the therapeutic effectiveness of these components, we plan to determine the anti-inflammatory effect, namely the expression of anti-inflammatory cytokines by lymphoid cells under the influence of ascorbic acid and lysozyme hydrochloride, in further studies.

### 3.4. Study of Ascorbic Acid and Lysozyme Hydrochloride Effects on Biofilm Formation

The study of the APIs effect on biofilm formation was carried out in 2 stages.

**Figure 7:**
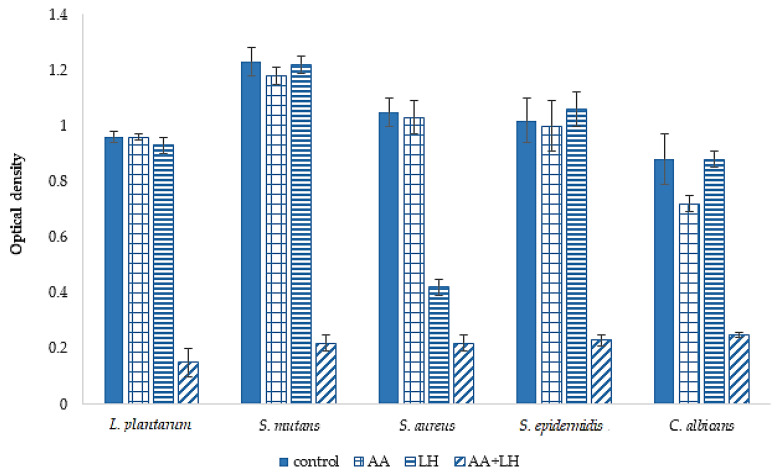
The ability of lysozyme hydrochloride (LH) and ascorbic acid (AA), separately and in combination, to prevent of microbial cultures biofilms formation, *n* = 3, *p* < 0.05.

The purpose of the first stage was to determine the influence of both the individual components of the chewing gum sample and their combined use on the process of biofilm formation. The results are presented in Figure 7.

The results in Figure 7 show that the use of lysozyme hydrochloride did not demonstrate a pronounced effect on the ability to prevent biofilm formation, under the influence of this API, only the optical density units of staphylococcal biofilms decreased by 2.5 times compared to the control results. The ability of ascorbic acid to prevent biofilm formation showed a less pronounced effect in comparison with lysozyme hydrochloride—the optical density of diurnal biofilms in all tested microbial cultures did not differ from the corresponding control indicators. At the same time, the results of the API combination effect evaluation on the studied indicator demonstrated a tendency to inhibit the formation of biofilms by the microorganisms involved in the experiment. Thus, it was established that the combined use of ascorbic acid and lysozyme hydrochloride was accompanied by a decrease in the optical density of diurnal *L. plantarum* biofilms by 6.4 times compared to the optical density of biofilm before the application of these API combinations (0.15 ± 0.05 and 0.96 ± 0.02 O.D., respectively), and *S. mutans* by 5.6 times compared to the control (0.22 ± 0.03 and 1.23 ± 0.05 O.D., respectively). Regarding representatives of staphylococcal microorganisms, the ability of the combined use of APIs to prevent biofilm formation was also determined: the optical density of diurnal *S. aureus* biofilms decreased by 4.7 times and that of *S. epidermidis* by 4.4 times compared to the control. The tendency to inhibit the formation of biofilms was also established for *C. albicans*—the optical density decreased by 3.5 times regarding the control.

**Figure 8:**
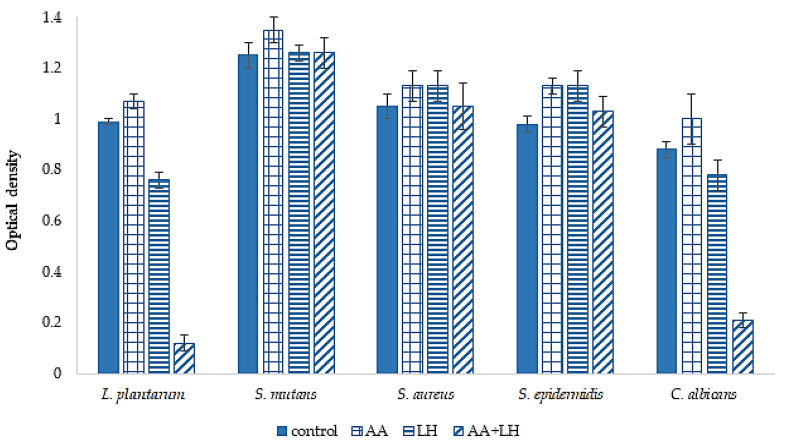
The ability of lysozyme hydrochloride (LH) and ascorbic acid (AA), separately and in combination, to destroy diurnal biofilms of microbial cultures, *n* = 3, *p* < 0.05.

The next stage of our research was the study of the active components ability to destroy biofilms of microorganisms. The results are given in Figure 8.

As shown in the results presented in Figure 8, the combination of ascorbic acid and lysozyme hydrochloride showed the ability to destroy the diurnal biofilms of *L. plantarum* and *C. albicans*, which exceeded the control results by 8.3 and 4.2 times, respectively. With regard to the diurnal biofilms of other microorganisms, it was established that the studied substances, both individually and in combination, do not reveal a statistically significant difference compared to the control.

## 4. Conclusions

In these pharmacological and microbiological research using various cultures of cells, the safety issue and effectiveness of the combined use of lysozyme hydrochloride and ascorbic acid (in concentrations of 10 mg and 20 mg, respectively) in compressed medicated chewing gum for prevention and treatment of dental diseases were investigated for the first time.

The individual and combination impact of active pharmaceutical ingredients on human embryonic kidney cells of the Hek293 line revealed no toxic antiproliferative effects, which may indicate a positive pharmacological action of the lysozyme hydrochloride and ascorbic acid combination in the studied therapeutic range on the cells of the oral cavity in destructive-inflammatory diseases of the periodontium and mucous membranes.

In the study of prooxidant-antioxidant homeostasis, which is manifested by the activation of lipid peroxidation processes and a decrease in the activity of antioxidant enzymes on cell cultures, it was confirmed that these active ingredients in the established concentrations promoted antioxidant protection and prevented the development of oxidative stress—one of the leading links in the development of inflammatory diseases of periodontal tissues. The results also showed that monocomponent therapy with each of the active substances did not have a significant effect on the studied parameter. On the contrary, their combined use increased the antioxidant action, which indicates potentiation and summation of the effects of lysozyme hydrochloride and ascorbic acid.

The morphology of the cells is not affected by lysozyme hydrochloride, ascorbic acid, or their combination, which confirms the safe choice of API concentrations and indicates the absence of side effects of dental chewing gums on cell viability.

Based on the microbiological studies, it was established that under the influence of the API combination of the medicated chewing gum, in contrast to their separate use, there is a tendency to suppress the biofilm formation of all studied microorganisms (bacteria *L. plantarum, S. mutans, S. aureus, S. epidermidis,* and fungi of the genus *Candida*), which also indicates the potentiation and summation of the active ingredients’ effects of the developed dental medicine. The combination of lysozyme hydrochloride and ascorbic acid also demonstrated a reliable ability to destroy diurnal biofilms of the bacteria *L. plantarum* and fungi of the genus *Candida*.

The obtained results, reflecting the biological behaviour of the tested active substances in combination and their positive influence on molecular mechanisms in living cells, will be considered in further pharmacological studies of the developed compressed medicated chewing gum.

## Figures and Tables

**Table 1:** MCGs formulation.

Name of Ingredient	Amount, mg/per Gum	Functions
Lysozyme hydrochloride	10.0	Active pharmaceutical ingredient
Ascorbic acid	20.0	Active pharmaceutical ingredient
Solo Sucralose Non-Micronised NF	1.5	Intense sweetener
Nat Apple Flavour Wonf	20.0	Taste additive
Apple FLV LQD FA-BO2980	6.0	Flavour
Syloid^®^ 244FP	10.0	Moisture scavenger, carrier for liquid flavour, glidant
Magnesium stearate	15.0	Lubricant
Health in Gum^®^ PWD 01	Up to 1000.0	Chewing gum base

**Table 2:** Assessment of biofilm formation by O.D. values.

O.D. Values	Adhesion to the Surface	Ability to Biofilms Formation
<0.12	Absent	Absent/weak
0.12–0.24	Average	Average
>0.24	Strong	High

## Data Availability

All data are available upon request.
